# Inhibition of AURKA kinase activity suppresses collective invasion in a microfluidic cell culture platform

**DOI:** 10.1038/s41598-017-02623-1

**Published:** 2017-06-07

**Authors:** Jiang-Long Xia, Wen-Jun Fan, Fei-Meng Zheng, Wen-Wen Zhang, Jia-Jun Xie, Meng-Ying Yang, Muhammad Kamran, Peng Wang, Hong-Ming Teng, Chun-Li Wang, Quentin Liu

**Affiliations:** 1 Institute of Cancer Stem Cell, Dalian Medical University, Dalian, China; 2 Department of Hematology, The Third Affiliated Hospital; Institute of Hematology Sun Yat-sen University, Guangzhou, China; 3 Department of Medical Oncology, The Eastern Hospital of The First Affiliated Hospital, Sun Yat-Sen University, Guangzhou, China; 4 Department of Oncology, The First Hospital Affiliated to Dalian Medical University, Dalian, China; 5 Department of Hematology, The Second Affiliated Hospital of Dalian Medical University, Dalian, China; 6 Department of Thoracic Surgery, The First Hospital Affiliated to Dalian Medical University, Dalian, China; 7 Sun Yat-Sen University Cancer Center, State Key Laboratory of Oncology in South China, Collaborative Innovation Center for Cancer Medicine, Guangzhou, China

## Abstract

Tumor local invasion is the first step of metastasis cascade which remains the key obstacle for cancer therapy. Collective cell migration plays a critical role in tumor invading into surrounding tissues. *In vitro* assays fail to assess collective invasion in a real time manner. Herein we aim to develop a three-dimensional (3D) microfluidic cell invasion model to determine the dynamic process. In this model, collective invasion of breast cancer cells is induced by the concentration gradient of fetal bovine serum. We find that breast cancer cells adopt a collective movement rather than a random manner when the cells invade into extracellular matrix. The leading cells in the collective movement exhibit an increased expression of an Aurora kinase family protein - AURKA compared with the follower cells. Inhibition of AURKA kinase activity by VX680 or AKI603 significantly reduces the phosphorylation of ERK1/2 (Thr202/Tyr204) and collective cohort formation. Together, our study illustrates that AURKA acts as a potential therapeutic target for suppressing the process of tumor collective invasion. The 3D microfluidic cell invasion model is a reliable, measurable and dynamic platform for exploring potential drugs to inhibit tumor collective invasion.

## Introduction

Tumor metastasis has caught extensive attention for decades since it explains 90 percent of tumor patients mortality^1^. During development, cells form cohesive groups to make and reshape tissues. The cohesive cell behavior is termed “collective migration”^2^. In the context of cancer, metastasis consists of sequential events which are known as the famous “invasion-metastasis cascade”. Invading locally through surrounding matrix and stromal cells is the first step of the whole procedure. For many cancer types, histomorphologically, the most frequently observed invasion unit is a cell group: cancer cells invade into stroma as cohesive units which are guided by several highly invasive cells. This process is normally termed “collective invasion”^3, 4^. Collective movement is a well-recognized mode of migration during embryogenesis and tissue restitution normally induced by important cues provided by structural and molecular organization of surrounding tissue^5^. Several basic principles are involved in this dynamic process: (1) the maintenance of cell-cell interaction, (2) the formation of polarized morphologies, (3) the organization of cytoskeleton, (4) the generation tractive force, (5) the degradation and remodeling of extracellular matrix^4^. The cells in the leading edge, termed “leader cells”, play an important guiding role in the dynamic process and normally exhibited mutual molecular characteristics different from the follower cells^6, 7^. In *Xenopus*, cephalic trunk neural crest cells start their migration as a cohesive cell population before progressively dissociating as single cells. Cells at the leading edge respond to an Sdf1 gradient by an N-cadherin/CIL-dependent mechanism^8^. During intestinal epithelial restitution, CXCR4 is activated by CXC chemokines ligand 12 (CXCL12) and stimulates the localization of E-cadherin. The activation of CXCL12/CXCR4 leads to a ROCK-dependent increase in F-actin accumulation in the collectively migration intestinal epithelial sheets, which promotes the paracellular space closure^9^. Mechanisms of collective cell migration in physiological processes can also be hijacked by malignant cancer cells during the tumor progression^10^. However, the mechanisms of collective cell movement in cancer are poorly studied.

*In vitro* models have conducted to simulate cellular behavior inside actual tumor microenvironment. However, the *in vivo* tumor microenvironment is more complicated than the *in vitro* tumor microenvironment, in which scaffolding structure is provided and cancer cells emerge different morphological and biological characteristics from two-dimensional (2D) culturing. Tumor metastasis is not only determined by the nature of malignant cancer cells themselves, but also by the interaction with surrounding stroma. However, traditional assays, including wound healing assay and transwell assay, can hardly reconstruct the spatiotemporal complexity. Meanwhile, the dynamic process of collective invasion in these traditional assays is difficult to evaluate^11^.

Microfluidic technology has become a well-established platform for studying cancer metastasis and invasion in 3D microenvironment^12^. Recently, a group develops a transendothelial invasion-model contained the basic components of blood vessels, such as vessel cavity, endothelium, and perivascular ECM containing chemokines. In this device, salivary gland adenoid cystic carcinoma (ACC) cellular aggregates transmigrated across the endothelium with the stimulation of CXCL12^13^. In another 3D *in vitro* microfluidic model, an osteo-cell conditional microenvironment is generated to analyze the extravasation of highly metastatic breast cancer cells into an organ-specific site^14^. Breast cancer cells responds to bone-secreted chemokine CXCL5 via surface receptor CXCR2 and extravasates through human umbilical vein endothelial cells (HUVECs) formed endothelial monolayer. The response of cancer cells to extracellular stimulate, which is critical in the initiation of tumor cell movement, can be dynamically monitored and quantified in microfluidic chips. Therefore, the microfluidic platform is a preferential model to study the behavior of tumor cell metastasis and to identify critical factors participating in this process.

AURKA belongs to Aurora kinase family, which plays a central role in mitosis^15^. Previous study indicates that AURKA also serves as an independent biomarker for survival in breast cancer^16^. The overexpression of AUKRA is related to the high risk of distant recurrence and involves in tumor metastasis^17^. In previous study, our group found that AURKA enhanced breast cancer cell migration by enhancing the dephosphorylation and activation of cofilin, which facilitated actin reorganization^18^. Meanwhile, AURKA was demonstrated to upregulate the expression of matrix metalloproteinase-2 (MMP2) by promoting the activity of p38 MAPK and Akt protein kinases in esophageal squamous cell carcinoma (ESCC)^19^. These studies suggest that AURKA could be a promising target for anti-metastasis treatment. However, whether AURKA involves in tumor local invasion remains elusive.

Numerous Aurora kinase inhibitors (AKIs) have been developed and been evaluated in both solid tumors and hematologic malignancies^20, 21^. Among them, clincal trials of MNL8237 have been carried out in patients with ovarian, peritoneal carcinoma and acute myelogenous leukemia and yielded promising outcomes^22–24^. In previous study we designed and synthesized a novel and potent Aurora kinase inhibitor, AKI603^20^. AKI603 inhibited AURKA activity in a small dose and overcome chemoresistance by targeting breast cancer initiating cells. Despite AKIs having the supporting experimental evidence in tumor prevention, whether AKIs could inhibit tumor cell local invasion in 3D microenvironment remains to be settled.

In this study, we developed a use-friendly microfluidic chip to mimic cell invasion process responding to serum gradient in real time. Using the microfluidic model, we revealed that AURKA was a critical regulator for collective invasion in the leader cells, which guided the formation of collective cohorts. Furthermore, ERK was highly activated in leader cells and phosphorylated in an AUKRA kinase dependent manner, which could be responsible for the high invasiveness of the leader cells. We demonstrated that targeting the kinase activity of AURKA could be a strategy to suppress collective cell invasion in the microfluidic model. Together, our study reveals a previously unknown function of AURKA in controlling collective invasion process, which might provide a promising strategy for overcoming cancer metastasis.

## Results

### Microfluidic chip design, fabrication and application in evaluating cell invasion

**Figure 1:**
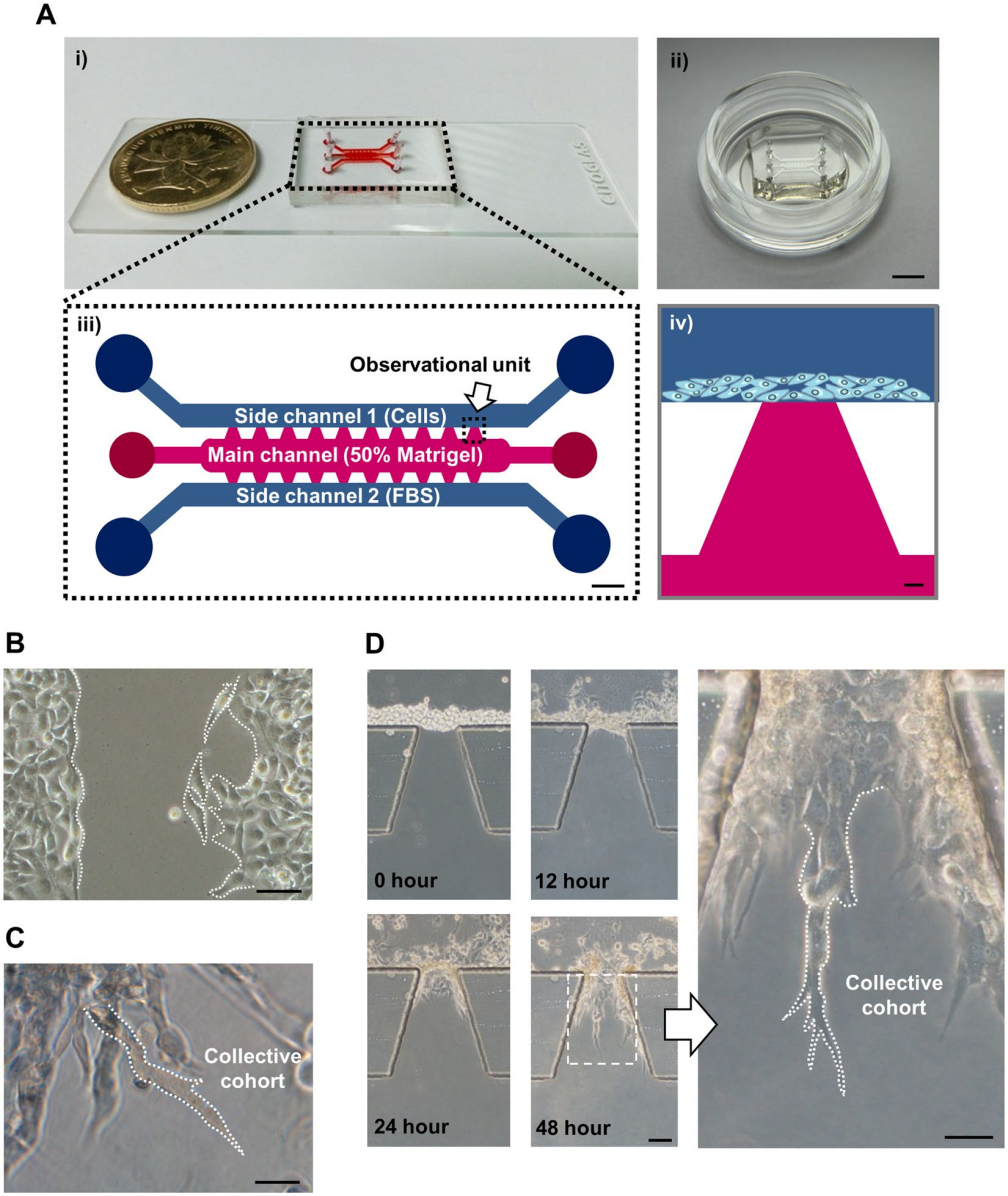
The fabrication of microfluidic chip and application for studying tumor invasion. (**A**) (i,ii) The images of microfluidic chip. Scale bar: 5 mm. (iii) Schematic of the microfluidic chip. The chip is composed of one central chamber (red) and two side channels (blue). The central chamber was filled with 50% matrigel. Cells with culture medium with 1% FBS were seeded in one of the side channels. Cell culture medium with 10% FBS was loaded to another side Chanel. A diffusion-based concentration gradient of FBS was formed between the side channels. Detailed parameters are illustrated in materials and methods. Scale bar: 500 μm. (iv) The observation unit. Cancer cells in cell culture medium (contains 1% FBS) are seeded in one of the side channels. The central chamber is filled with 50% matrigel. Serum gradient is generated by the 10% FBS containing cell culture medium injected into the opposite side channel. Scale bar: 100 μm. (**B**) Morphology of MDA-MB-231 cells in wound healing assay. (**C**) Morphology of MDA-MB-231 cells in 3D culture. (**D**) MDA-MB-231 cells were seeded in the side channel of the microfluidic chip. Images were taken at 0 hour, 12 hours, 24 hours and 48 hours respectively. Scale bar: 100 μm. Arrow indicates the highlight of morphology of cell collective cohorts. Scale bar: 50 μm.

To evaluate the invasion of tumor cells, we designed and fabricated a microfluidic device for local invasion study based on previous report (Fig. 1A)^14^. This model was comprised of two side channels and one main chamber. The side channels connected with the main chamber via 10 parallel trapezoidal galleries. The galleries were designed into trapezoidal to form a ‘stop fluid’ structure which generated a smooth and evident interface between cell culture medium and Matrigel. To mimic the tumor bulk surrounded by cohesive stromal matrix, cells were enriched and laid on Matrigel surface. Meanwhile, culture medium containing high concentration serum was introduced into the opposite channel and changed promptly to form a stable serum gradient across the vertical axis of the observational unit^13, 25^. Cell invasive behaviors responding to the serum gradient can be closely monitored inside this platform, since poly-dimethyl-siloxane (PDMS) layer is oxygen permeable and translucent.

**Figure 2:**
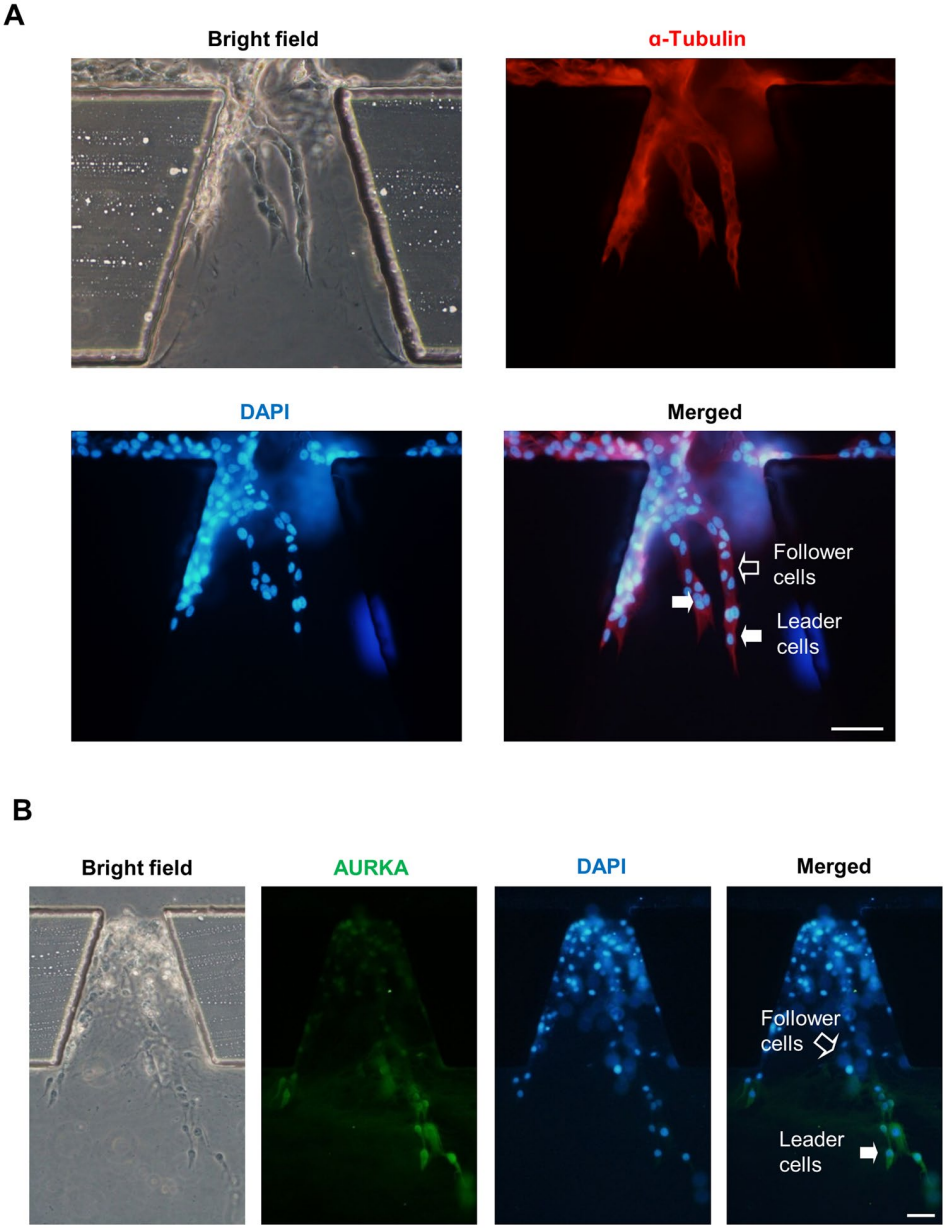
The different expression levels of AURKA in the leader cells and the follower cells. (**A**,**B**) MDA-MB-231 cells were cultured in microfluidic chip for 2 days and subjected to immunofluorescence staining and analysis (original magnification, ×200) with antibodies against α-Tubulin (red, **A**) and AURKA (green, **B**). DAPI (blue) staining was used to visualize the nuclei. Solid arrows point to the leader cells while the hollow arrow points to the follower cells. Scale bar: 100 μm (**A**) and 100 μm (**B**).

Next, we identified the morphological characteristics of MDA-MB-231 cells in different metastasis models. In a wound healing assay, cells without restriction of hydrogel scaffold moved across scratch in an inattentive structure without specific direction (Fig. 1B). In a classic 3D cell culturing model, cells were embedded within high concentration of Matrigel (Fig. 1C). Cells spread out in a radical pattern due to the internal pressure and external relatively high concentration nutrition. The morphology of strands was consistent with previous description on collective cohort (labeled in Fig. 2B)^4^. MDA-MB-231 cells were as well cultured inside the chip (Fig. 1D). In the first 12 hours, cells attached to the Matrigel and extended protrusions, which was induced by serum gradient generated from culture medium in the opposite side channel. By the second day of culturing, the cells migrated in a stalk-like pattern, rather than a random manner, to form collective cohort. This was consistent with the observation from living cell station in real time manner (Supplementary Movie 1).

### Leader cells guide collective invasion in a 3D hydrogel scaffold and expressed high level of AURKA

After inducing by FBS gradient for 2 days in a horizontal direction, MDA-MB-231 cells were subjected to immunofluorescence staining and laser scanning confocal microscope analysis combined with 3D reconstruction (Fig. 2A, Supplementary Movie 2, 3). We found that collective cohorts formed by MDA-MB-231 cells were well-organized cohesive multicellular units inside Matrigel, rather than a loose and individual structure. The follower cells followed the track exploited by leader cells along the long axis of the observation unit (Supplementary Fig. S1). This orderly and directive pattern led by the leader cells was more effective for tumor cells to invade^26^. To evaluate AURKA expression level in invasive strands, MDA-MB-231 cells were fixed after culturing for 4 days, and AURKA was detected by immunofluorescent staining (Fig. 2B). Result showed that MDA-MB-231 cells exhibited heterogeneity in AURKA expression level, and the leader cells carried more AURKA protein than the follower cells, suggesting that AURKA might be critical for the function of leader cells during collective invasion.

### The expression of AURKA is critical for the formation of collective cohorts

**Figure 3:**
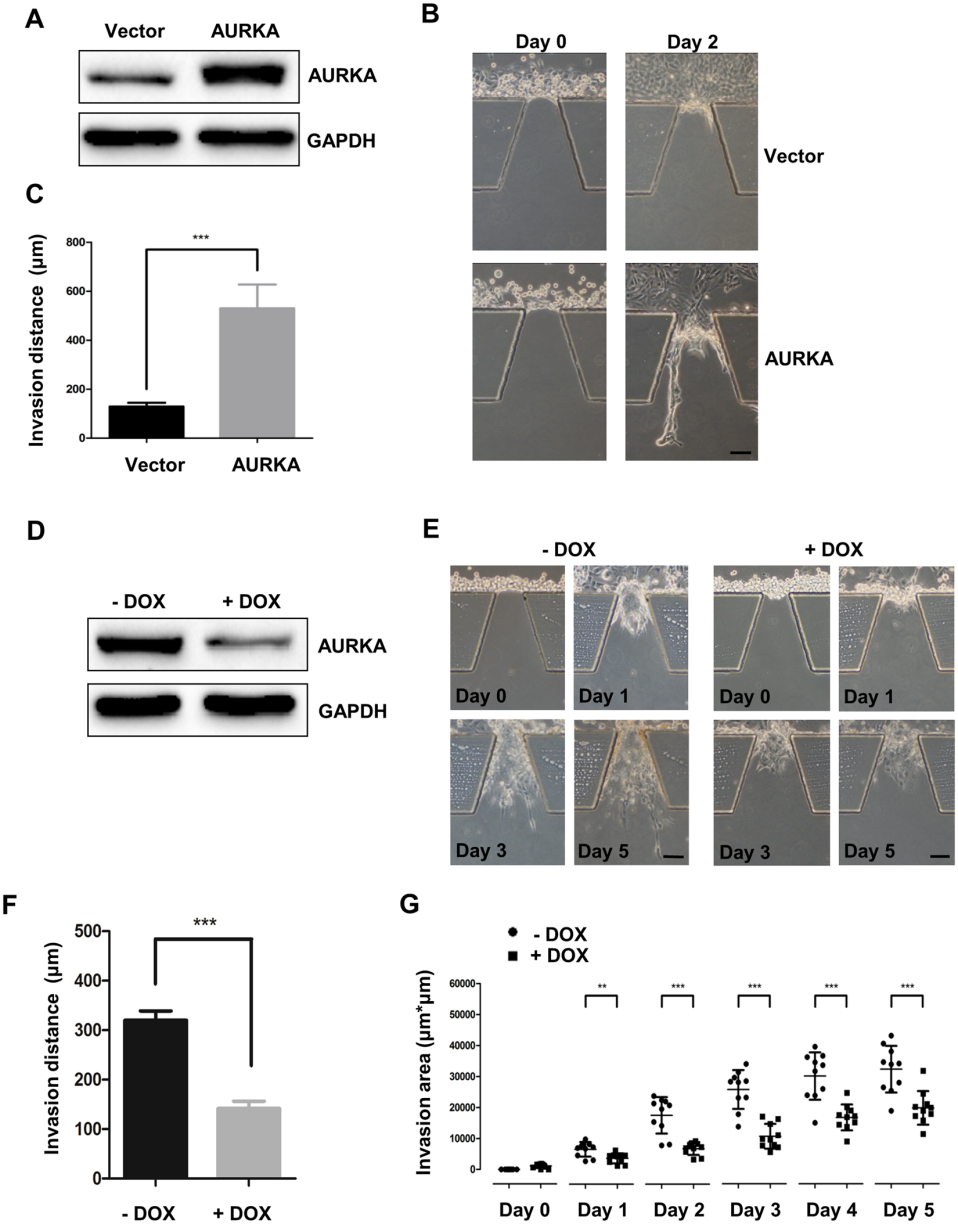
AURKA regulates collective cohort formation during tumor cell invasion. (**A**) AURKA overexpressed or control MCF-10A cells were subjected to western blot analysis. (**B**) AURKA overexpressed or control MCF-10A cells were seeded in microfluidic chip. Images were taken at day 0 and day 2 respectively. Scale bar: 100 μm. (**C**) Invasion distance of collective cohorts at day 2 in 10 duplicated galleries were counted and were presented as the means ± SD (***P < 0.001, Student t test). (**D**) Endogenous AURKA was conditional knocked down via treating with 0.2 μM doxycycline in MDA-MB-231^tet-on shAURKA^ cells. (**E**) MDA-MB-231^tet-on shAURKA^ treated with or without doxycycline were seeded in microfluidic chip. Images were taken at day 0, day 1, day 3 and day 5 respectively. Scale bar: 100 μm. (**F**) Invasion distance at day 5 were measured. Data summarized 10 duplicated galleries and were presented as the means ± SD (**P < 0.01, Student t test). (**G**) Invasion areas were calculated at day 0 to day 5 respectively (**P < 0.01; ***P < 0.001 Student t test).

Next, we overexpressed AURKA in a noncancerous breast epithelial cell line, MCF-10A (Fig. 3A). After seeding the AURKA overexpressing cells (MCF-10A-AURKA) and control cells (Vector) in microfluidic chip for 2 days, MCF-10A cells expansion can be identified in the side channels. However, MCF-10A cells seldom invaded into Matrigel in vector group. By contrast, MCF-10A-AURKA cells invaded into the Matrigel with the induction of FBS gradient, and formed invasive strands after 2 days culturing (Fig. 3B,C). Furthermore, AURKA promotes collective invasion via a MMP-dependent manner (Supplementary Fig. S2). This result showed that AURKA was crucial for MCF-10A cells to overcome the obstacle of extracellular and to invade in a collective manner, which was consistent with its high expression in the leader cells.

To validate the role of AURKA in cancer cell invasion, we built up a tet-on shRNA system to down-regulate AURKA expression in MDA-MB-231 cells (shAURKA MDA-MB-231) (Fig. 3D). We cultured shAURKA group and control group in microfluidic chips for five days (Fig. 3E). The invasion distance determined by the length of collective cohorts was effectively inhibited via knocking down AURKA at day 5, and the invasion area was also reduced (Fig. 3F,G).

These results indicated that AURKA promoted the formation of collective cohorts, which were driven by high invasive leader cells. Meanwhile, AURKA not only played a crucial role in the collective invasion in MDA-MB-231 cells, but also in the A549 cells (lung cancer cell line) and AGS cells (gastric cancer cell line) (Supplementary Fig. S3). The invasive ability was reduced when AURKA was knocked down in these two cell lines, consistent with the results from transwell assay. These results indicated that AURKA not only promoted collective invasion in breast cancer, but also in other type of cancers.

### ERK acts as a downstream of AURKA during local collective invasion

**Figure 4:**
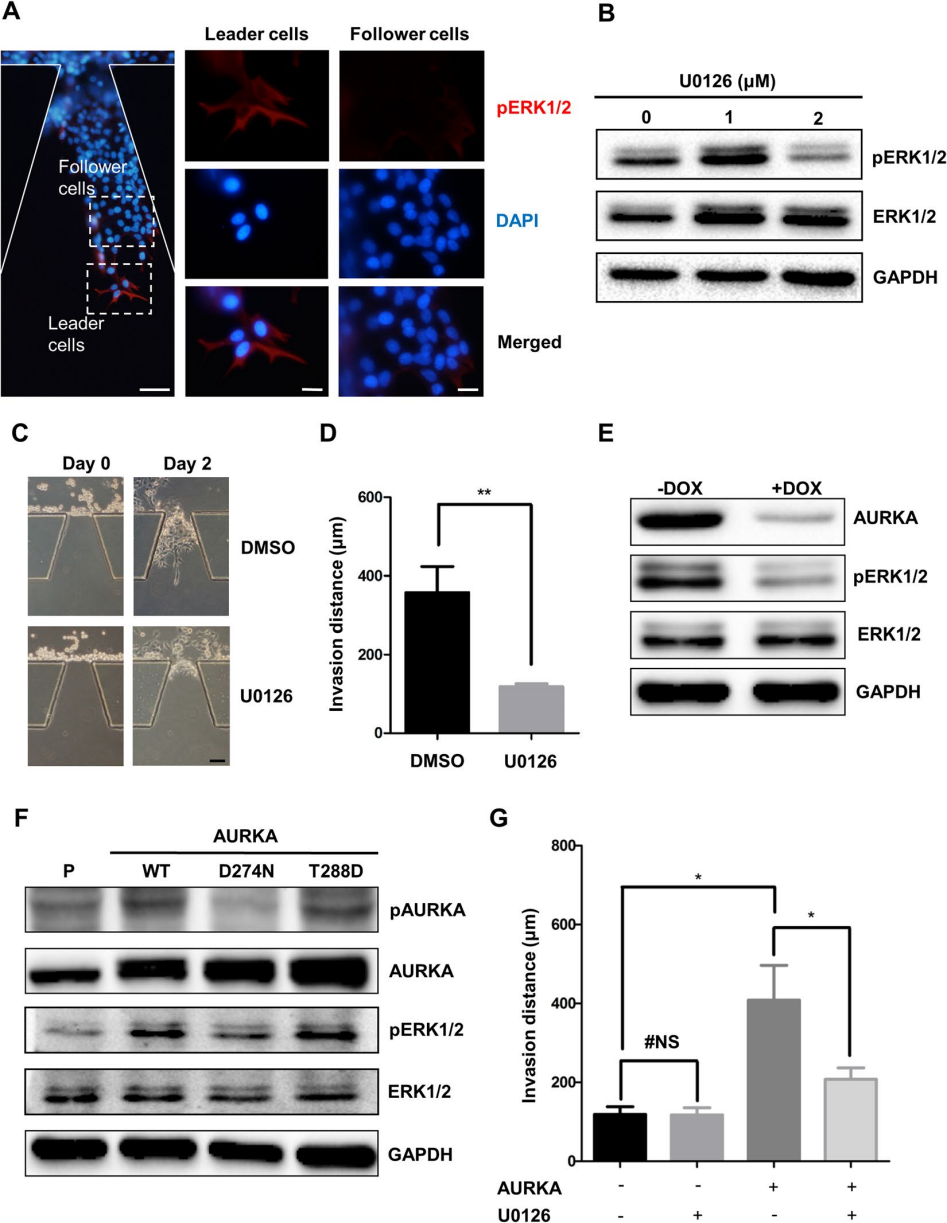
Erk1/2 is necessary for AURKA promoted collective invasion. (**A**) MDA-MB-231 cells were cultured in microfluidic chip for 2 days and subjected to immunofluorescence staining and analysis (original magnification, ×100, scale bar: 50 μm) with antibodies against pErk1/2 (red) and DAPI (blue) staining was used to visualize the nuclei. Leader cells and follower cells were highlighted respectively (original magnification, ×400, scale bar: 20 μm). (**B**) MDA-MB-231 cells treated with U0126 for 2 days were subjected to western blot analysis of with Erk1/2, pErk1/2 and GAPDH antibodies. (**C**) MDA-MB-231 cells treated with or without U0126 were seeded in microfluidic chips. Images were taken at day 0 and day 2 respectively. (**D**) Invasion distance of collective cohorts at day 2 in 10 duplicated galleries were counted and were presented as the means ± SD (**P < 0.01, Student t test). (**E**) Cell lysates of MDA-MB-231^tet-on shAURKA^ cells treated with 0.2 μM doxycycline and control were analyzed by western blot analysis of AURKA, pERK1/2, ERK1/2 and GAPDH antibodies. (**F**) Cell lysates of MCF-10A^P^, MCF-10A^wt AURKA^, MCF-10A^D274N AURKA^, MCF-10A^T288D AURKA^ were subjected to western blot analysis of pAURKA, AURKA, pERK1/2, ERK1/2 and GAPDH antibodies. (**G**) MCF-10A^P^, MCF-10A^wt AURKA^ cells and U0126 treated MCF-10A^P^ and MCF-10A^wt AURKA^ cells were seeded in microfluidic chips. Invasion distances were counted (*P < 0.05, two-way ANOVA).

ERK is reported to regulate actin rearrangement and subsequent filopodia and lamellipodia extensions and regard as one of the dominant factors during cell collective movement^27, 28^. Therefore, we evaluated the distribution of p-ERK (Thr202/Tyr204) among leader cells and follower cells of collective cohort. After two days culturing, MDA-MB-231 cells were fixed and subjected to immunofluorescence staining, and leader cells and follower cells were circled according to their relative positions (Fig. 4A). As shown in Fig. 4A, the cells at the leading edge of collective invasive strand expressed high level of phosphorylated ERK1/2. The enrichment of phosphorylated ERK1/2 was consistent with the distribution of AURKA. Next, the specific inhibitor of ERK, U0126, was utilized to inhibit the activity of ERK1/2 in MDA-MB-231 cells. The inhibition of ERK kinase activity was determined by western blot (Fig. 4B). We found that the collective invasion was effectively suppressed when the cells were treated with U0126 (Fig. 4C,D). ERK-Rac1 signaling coordinately regulates cancer cells motility. To determine whether AURKA promotes collective invasion via ERK-Rac1 pathway, we treated the AURKA overexpressed MDA-MB-231 cells with Rac1 inhibitor. Collective invasion of MDA-MB-231 cells were blocked after Rac1 inhibitor treatment (Supplementary Fig. S4). These results suggested that the activity of ERK1/2 is involved in AURKA regulated collective invasion. Consistent with our hypothesis, knocking down of AURKA significantly decreased the phosphorylation level of ERK1/2 in MDA-MB-231 cells (Fig. 4E). We further examined whether AURKA kinase activity was important for ERK activation. Plasmids expressing wild type (WT), kinase dead (D274N) and kinase activated (T288D) form of AURKA and empty vector were stably transfected in MCF-10A cells. The overexpression of AURKA and the autophosphorylation of AURKA Thr288, which reflect the activation of AURKA, were confirmed (Fig. 4F)^29, 30^. Interestingly, the activity of ERK was increased in cells expressed kinase activated AURKA compared with cells expressed wild type AURKA, whereas the activity of ERK was decreased in cells expressed kinase dead AURKA compared with cells expressed wild type or kinase activated AURKA. These results implied that ERK might function as a downstream of AURKA.

Due to AURKA functioning as an upstream factor of ERK, we evaluated whether inhibition of ERK activation might suppress AURKA promoted collective invasion. By treating with 2 μM U0126 for 2 days, the invasion distance promoted by exogenous AURKA was reversed in MCF-10A cells (Fig. 4G, Supplementary Fig. S5), suggesting that ERK acted as a downstream of AURKA to modulate collective cohorts formation during tumor local invasion.

### AURKA small molecular inhibitors reduce collective invasion

**Figure 5:**
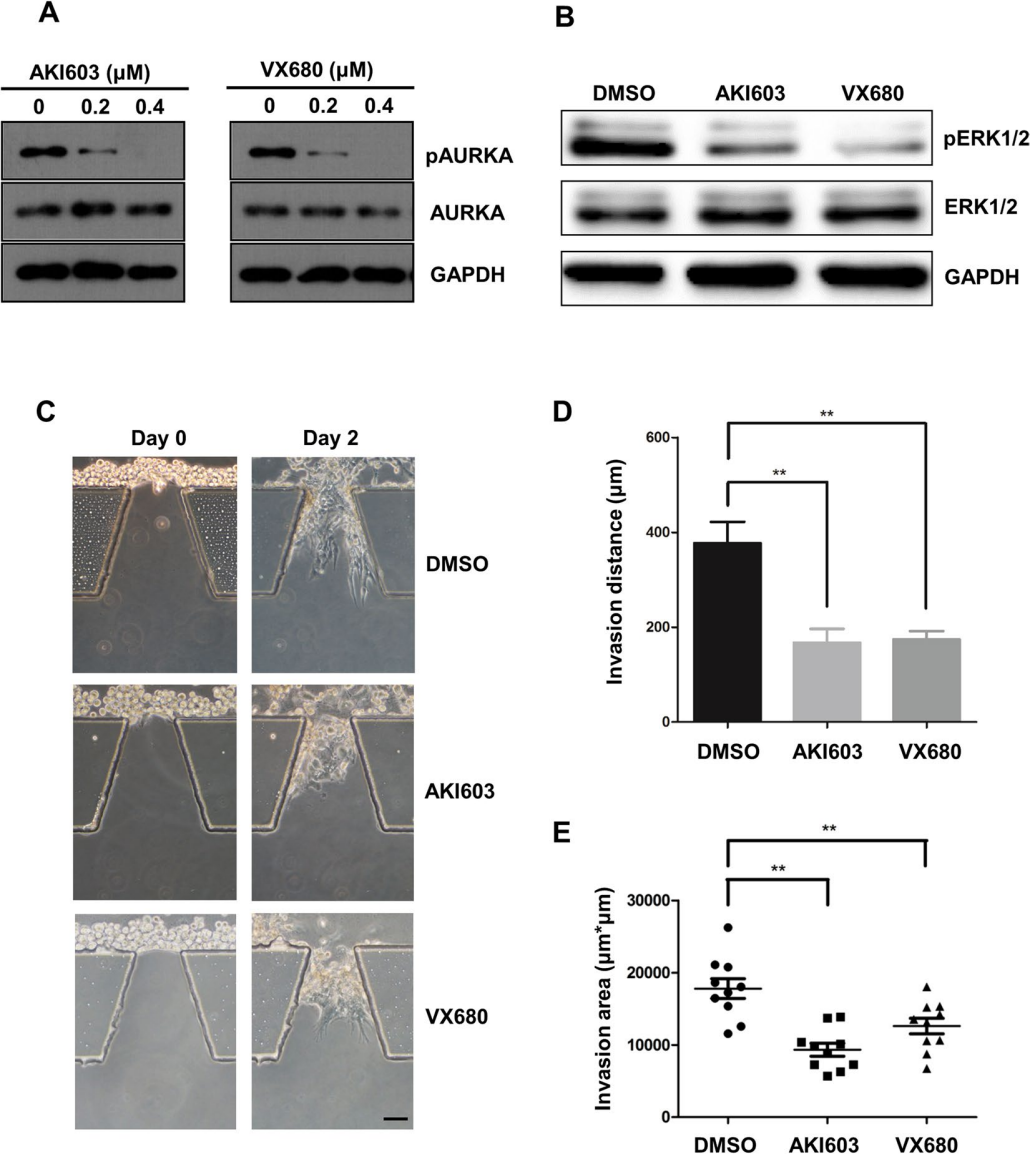
Inhibition of AURKA kinase activity by small molecular inhibitors reduces collective invasion. (**A**) MDA-MB-231 cells treated with AKI603 and VX680 for 2 days were subjected to western blot analysis of pAURKA, AURKA and GAPDH antibodies. (**B**) MDA-MB-231 cells treated with DMSO, 0.2 μM AKI603 and 0.2 μM VX680 were subjected to western blot analysis of pErk1/2, Erk1/2 and GAPDH antibodies. (**C**) MDA-MB-231 cells treated with DMSO, 0.2 μM AKI603 or 0.2 μM VX680 were seeded in microfluidic chips. Scale bar: 100 μm. (**D**,**E**) Invasion distance and invasion area were calculated after culturing for 2 days (*P < 0.05; **P < 0.01, two-way ANOVA).

Next, we examined whether Aurora kinase inhibitors suppress the collective invasion in this system. In previous study, we synthesized a potent Aurora kinase inhibitor, AKI603, which showed a high anti-cancer effect in breast cancer. MDA-MB-231 cells were treated with different concentrations of AKI603 or VX680 for 2 days. The AURKA kinase activity was significantly inhibited at 0.2 μM both with AKI603 or VX680 (Fig. 5A). The phosphorylation of ERK1/2, as downstream of AURKA, was also significantly suppressed in MDA-MB-231 cells when treated with 0.2 μM AKI603 or VX680 (Fig. 5B).

To test whether the collective invasion can be reduced by inhibiting AURKA kinase activity in the microfluidic model, the collective invasion was evaluated in MDA-MB-231 cells treated with AKI603 or VX680 for 2 days (Fig. 5C). The wild type MDA-MB-231 cells invaded into Matrigel with the induction of FBS gradient. On the contrary, the invasion distance and overall invasion area were effectively inhibited in AKI603 and VX680 groups (Fig. 5D,E). These results suggested that AKI603 and VX680 effectively suppressed tumor collective invasion in a 3D environment. Furthermore, the 3D microfluidic cell invasion model was a potent model to evaluate the effects of small molecular inhibitors on tumor local collective invasion.

## Discussion

In this study, we built up a 3D microfluidic cell invasion model to mimic the local invasion of tumor cells. MDA-MB-231 cells degraded the Matrigel and made the way towards the gradient of FBS. We showed that the tumor cells invade into surrounding matrix in a 3D collective manner rather than a random manner (Fig. 1B,C). The collective cohorts were guided by a small amount of highly invasive subpopulation called “leader cells”. AURKA was highly expressed in the leader cells, and responsible for their high motility (Fig. 2B). The phosphorylation of ERK1/2 was enhanced by AURKA in leader cells and was suppressed when the activity of AURKA was inhibited by VX680 or AKI603 (Figs 4 and 5B). Furthermore, our data showed that inhibition of AURKA kinase activity by low dose of AKI603 and VX680 significantly suppressed cell collective invasion (Fig. 5C–E).

Collective cell migration is pivotal in regulating both physiological and pathological biological process, such as wound healing and cancer metastasis. The cells do not migrate individually but collectively either as clusters, chains, or sheets. Collective migration is found to keep the tissue intact during remodeling, allows mobile cells to carry other cells that are otherwise immobile. In cancers, this process is guided by a certain small amount of high invasive cells, named “leader cells”. Interestingly, the collective invasion of tumor cells is only observed in 3D ECM microenvironment. This suggests that leader cells are responsible for the tumor cells invading into ECM scaffold and could be ideal targets for anti-metastasis therapy. Therefore, identifying the molecular mechanisms of tumor cell collective invasion would shed light on targeted therapy^27^.

Traditional experimental models used for evaluating tumor cell migration or invasion include transwell assay, wound healing assay and 3D cell culture embedded in Matrigel. Transwell assay is normally used to test the response of tumor cells to chemokines and inhibitors, which is also known as the Boyden chamber assay. To conduct Transwell assay, the migrated cells are stained and counted. The membrane is usually coated with ECM component for invasion study. In this assay, the observation is restricted to a certain time point. The morphological characteristics of invasion are unable to demonstrate, and the dynamic changes cannot be quantified. The wound healing assay is an alternative model to assess tumor cell migrating ability. The migration of confluent cells across a flat surface into a space generated by pipette tips was observed, and the motility of cells was evaluated by measuring the shorten distance. Disadvantages of wound healing assay include restriction to a 2D culturing mode. Cell migrated across the space in a broad cell sheets rather than an organized network, since no extracellular component is considered in this model. With recent advancements in micromaching, optics and chemistry, research group have implement the wound healing assay in a well-controlled manner^11^.

3D cell culture is a well-established model to investigate multicellular structure and function. The typical 3D cell culture assay is developed into a spheroid invasion assay to study tumor cell collective migration. Cell clusters are harvested and embedded into 3D ECM with or without organotypic chemokines, allowing the cell strands to stretch radially^6, 31^. Another approach belongs to 3D ECM-based assays is Vertical 3D ECM invasion culture. In this assay, multi-cell layers are grown above or below a 3D ECM scaffold (such as collagen I or Matrigel). The extent of collective cell invasion is measured as cell number and vertical penetration depth using microscopy and 3D confocal analysis^32^. One of the disadvantages of this approach is the inconvenient operation, poor maintenance and management. A number of factors should be carefully controlled when using this model. Furthermore, the cells invaded into the hydrogel in a vertical direction, which parallels with observation. The application of this model in continuous dynamic observation on invasive morphology is limited.

Microfluidic chips have been utilized in molecular biology and cellular biology for more than ten years, since the PDMS used in the chips fabrication is non-cytotoxic and air permeable^33^. Both of these characteristics are required for the molecular and functional assays. The microfluidic chip is a potent platform for collective invasion study, since tumor cells invade into ECM in a horizontal direction. The invasion process can be easily verified and quantified. Most of all, the invasion process simulated in this platform is closer to the *in vivo* local invasion.

In this platform, we validated that MDA-MB-231 cells invade into Matrigel as collective cohorts. These cohorts were led by small group of cells which displayed different phenotypic background from the followers. Interestingly, AURKA kinase activity was involved in collective invasion. We identified that AURKA is highly expressed in the leader cells during tumor collective invasion, which is responsible for the local invasion of malignant cells. The overexpression of AURKA in the leader cells activated ERK1/2, whose elevated activity has been identified as a marker of leader cells identity^27^. The up-regulation of ERK activity resulted in cell skeleton remodeling, motility increasing and secretion of ECM degrading proteins^28^. Meanwhile, the ERK activity of leader cells was required for regulating cellular speed which drove the forward migration of collective cohorts^34^. These characteristics endow leader cells the ability to invade into surrounding matrix under stimulation induced by the gradient of chemotactic factors, leaving a track of following cells to invade along with them.

During cell culturing in microfluidic chip, we found that tumor cells squeezed into hydrogel with no apparent leading edges at first day and exhibited as collective cohorts mostly at the second day after cell seeding. We hypothesis that the evident different morphologies after one day culturing are based on four reasons: (1) tumor cells were not fully contact with hydrogel at first day; (2) there were natural delay between the activation of cell signaling pathways and changes on cellular functional level; (3) highly invasive cells distributed randomly at first and need time to move to the leading edge; (4) the MMPs secreted by leader cells need to be accumulated to an adequate concentration to induce the remodeling of extracellular matrix.

Herein, we applied a novel small inhibitor of AURKA, AKI603, to inhibit collective cell invasion. AKI603 showed potent effects on cell invasion (Fig. 5C,D,E). These results suggest that AKI603 functions as a therapeutic agent to target tumor local invasion. Our data uncovered that the high expression of AURKA enhanced the leader cells guided collective invasion by enhancing the phosphorylation of ERK1/2 (Thr202/Tyr204). AKI603, as effective as VX680, is a promising inhibitor in suppressing breast tumor cell collective invasion.

Together, the new 3D microfluidic chip provides a use-friendly platform to text small inhibitors against tumor collective invasion. Meanwhile, we identified that AURKA is highly expressed in leader cells during collective invasion. Inhibition on AURKA kinase activity effectively suppresses invasion distance induced by leader cell and overall invasion area.

## Methods

### Microfluidic chips fabrication

The microfluidic device adopted in the present study consisted of one central chamber and two side channels. The main channel is 5000 μm long, 1000 μm wide and 80 μm high. Each side channel is 6000 μm long, 500 μm wide and 80 μm wide. Inlet and outlet ports of the PDMS (poly-dimethyl-siloxane; Silgard 184, Dow Chemical) devices were bored using disposable biopsy punches, and the PDMS layer was bonded to a cover glass or a petri dish to create 80 mm high microfluidic channels with oxygen plasma treatment. These devices were subsequently sterilized by UV or autoclave. Matrigel (BD Biosciences) was mixed with same volume cell culture medium and was injected into the central channel using a 200 μl pipette. The chips were placed in the 10 cm petri dishes which contain 3 ml sterile water and were ready for using after 20 minutes standing.

50% Matrigel was identified as convenient to process and utilized in the follows assays. After filling hydrogel into the central chamber, MDA-MB-231 cells suspension was injected into one of the side channels. Tumor cells were settled on the hydrogel surface via incubating the chip in an upright position in the incubator for 20 minutes. Then, the chip was returned to its original position. To set a directional introduction, tumor cells were suspended in 1% FBS containing cell culture medium while 10% FBS containing cell culture medium was introduced into the opposite channel.

### Cell culture in microfluidic device

MDA-MB-231, MCF-10A, A549 and AGS cell lines were used in this study. These cells were purchased from American Type Culture Collection (ATCC). All the cell lines were cultured at 37 °C with 5% CO_2_ and 95% relative humidity. MDA-MB-231 cells were cultured in DMEM (Gibco) supplemented with 10% fetal bovine serum (FBS, Hyclone). A549 and AGS were cultured in RPMI Medium 1640 supplemented with 10% FBS. MCF-10A was cultured in DMEM/F12 (1:1) supplemented with 5% horse serum, 20 ng/ml EGF, 100 ng/ml cholera toxin, 0.1 mg/ml insulin, 500 ng/ml hydrocortisone.

### Three-dimensional culture

Eight-chambered RS glass slides (BD Falcon) were precoated with 40 μL/well of Matrigel (BD Biosciences). The Matrigel was solidified at 37 C for 30 minutes. Next, the cells were suspended in growth medium containing a final concentration of 2% Matrigel and plated at a density of 2,500 cells per well. The cells were fed with growth medium containing 2% Matrigel every 4 days. After 10 days of culture, the cells were photographed using an inverted microscope.

### Immunofluorescence staining

The MDA-MB-231 cells cultured in microfluidic chips for 2 days were fixed in paraformaldehyde (PFA) for 20 minutes at 4 °C and were permeabilized in 0.5% TritonX-100 in PBS containing 0.03% BSA at room temperature for 10 minutes. The cells were then incubated with 1% BSA for 1 hour at room temperature to block nonspecific binding before the addition of the primary antibody. The chips were incubated with the primary antibody to α-Tubulin (mouse; Thermo, 1:200, catalogue number A11126), AURKA (rabbit; Millipore, 1:200, catalogue number 07–648), phospho-Erk1/2 (Thr202/Tyr204; Cell Signaling, 1:100, catalogue number 9101) at room temperature for 1 hour, followed by an Alexa Fluor 488 or 546-conjugated secondary antibodies (Invitrogen). After counterstaining with DAPI (1 μg/ml, Sigma), the cells were visualized using a fluorescence microscope (Olympus).

### Quantitative analysis of total length of collective cohorts and invasion areas

Invasion distance were defined as the distance between the starting gel face and the leading edge^12^. The method to quantify invasion area was established based on previous studies^22–24^. Invasion areas were defined as the areas surrounded by the invasive front edge, the starting gel surface and two bevel edges of neighboring trapezoids (Supplementary Fig. S6). Statistical analyses were performed with ImageJ program (available at http://rsb.info.nih.gov/ij/) and GraphPad Prism software (La Jolla, CA, USA).

### Transwell assay

After 72 hours doxycycline induction, cells were cultivated in serum-free medium for 12 hours. Cells were washed once in PBS, detached with trypsin/EDTA solution, and resuspended in relevant culture mediums. Then 2–5 × 10^5^ cells were seeded in the Matrigel-coated insert of a Boyden chamber (BD Biosciences). The lower chamber was filled with 750 μl of medium containing 10% FBS to induce chemotaxis. 24 to 48 hours later, the non-migrated cells in the upper chamber were gently scraped away, and adherent cells present on the lower surface of the insert were fixed with methanol, stained with PI, and counted in five randomly chosen fields from each chamber. Results were analyzed by Image J.

### Western blot analysis

The cells treated with indicated conditions were harvested and were lysed in RIPA buffer. The protein concentrations were determined by the Bradford method using BSA (Sigma) as the standard. Equal amounts of cell extract were subjected to electrophoresis in SDS–polyacrylamide gel and were transferred to nitrocellulose membrane (Millipore). The membranes were blocked and then incubated with primary antibodies at 4 °C overnight. The following primary antibodies were used: AURKA (rabbit; Millipore, 1:4,000, catalogue number 07–648), phospho-AURKA (T288; Cell Signaling, 1:1000, catalogue number 3079), Erk1/2 (Cell Signaling, 1:2000, catalogue number 9102), and phospho-Erk1/2 (Thr202/Tyr204; Cell Signaling, 1:2000, catalogue number 9101). Next, the membranes were incubated for 1 hour at room temperature with the appropriate secondary antibodies.

### Lentivirus preparation and transfection

Lentivirus was produced in 293 T cells using the second-generation packaging system plasmids psPAX2 (Addgene) and pMD2.G (Addgene). One 10 cm culture dish containing 5 × 10^6^ 293 T cells was transfected using Lipofectamine 2000 (Life) with 12 mg lentiviral vector, 9 mg psPAX2 and 3 mg pMD2.G. Supernatants were collected every 24 hours between 24 and 72 hours after transfection, pulled together and concentrated via ultracentrifugation, and the viral titre was determined by serial dilutions. The multiplicity of infection during transfection was 5.

### Statistical analysis

Statistical analyses were performed using the SPSS software, version 16.0 (Chicago, I L, USA) or with GraphPad Prism 5.0 (La Jolla, CA, USA). The unpaired Student *t* test was used to perform a statistical comparison between two groups.

## Electronic supplementary material

Supplementary Movie 1

Supplementary Movie 2

Supplementary Movie 3

Supplementary Figures
