# Supplementary material for: Inhibition of AURKA kinase activity suppresses collective invasion in a microfluidic cell culture platform
Source: Sci Rep. 2017 Jun 7;7:2973. doi: 10.1038/s41598-017-02623-1 (PMC5462816; doi:10.1038/s41598-017-02623-1)
Supplement: Supplementary file 4 — Supplementary Figures [file 41598_2017_2623_MOESM4_ESM.pdf]

Inhibition of AURKA kinase activity suppresses collective invasion in a microfluidic cell culture platform

Jiang-Long Xia, Wen-Jun Fan, Fei-Meng Zheng, Wen-Wen Zhang, Jia-Jun Xie, Meng-Ying Yang, Muhammad Kamran, Peng Wang, Hong-Ming Teng, Chun-Li Wang, and Quentin Liu

Supplementary results

S1

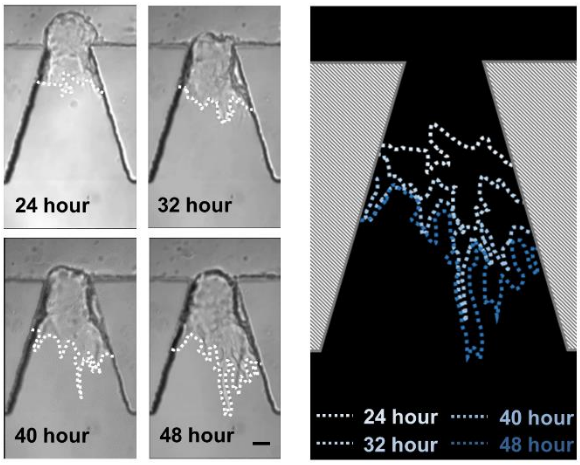

S1: Time-lapse microscopy images of MDA-MB-231 in microfluidic chips. (A) MDA-MB-231 cells were seeded in microfluidic device and images were obtained at indicated time points (24, 32, 40 and 48 hours). Overlapping of time-lapse images represents trajectories of leader and follower cells. Scale bar: 100 μm

S2

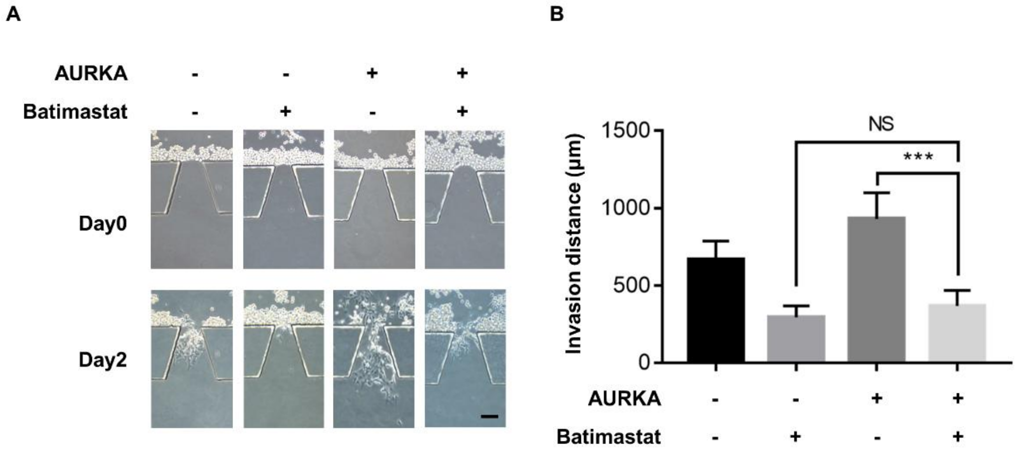

S2: AURKA promotes collective invasion via a MMP-dependent manner. (A) MDA-MB-231 cells were transfected with control or AURKA vectors cultured in microfluidic chip with or without Batimastat treatment. (B) Invasion distance at day 2 were measured. Scale bar: 100 μm. Data summarized 10 duplicated galleries and were presented as the means ± SD (**, $P < 0.001$, two-way ANOVA).

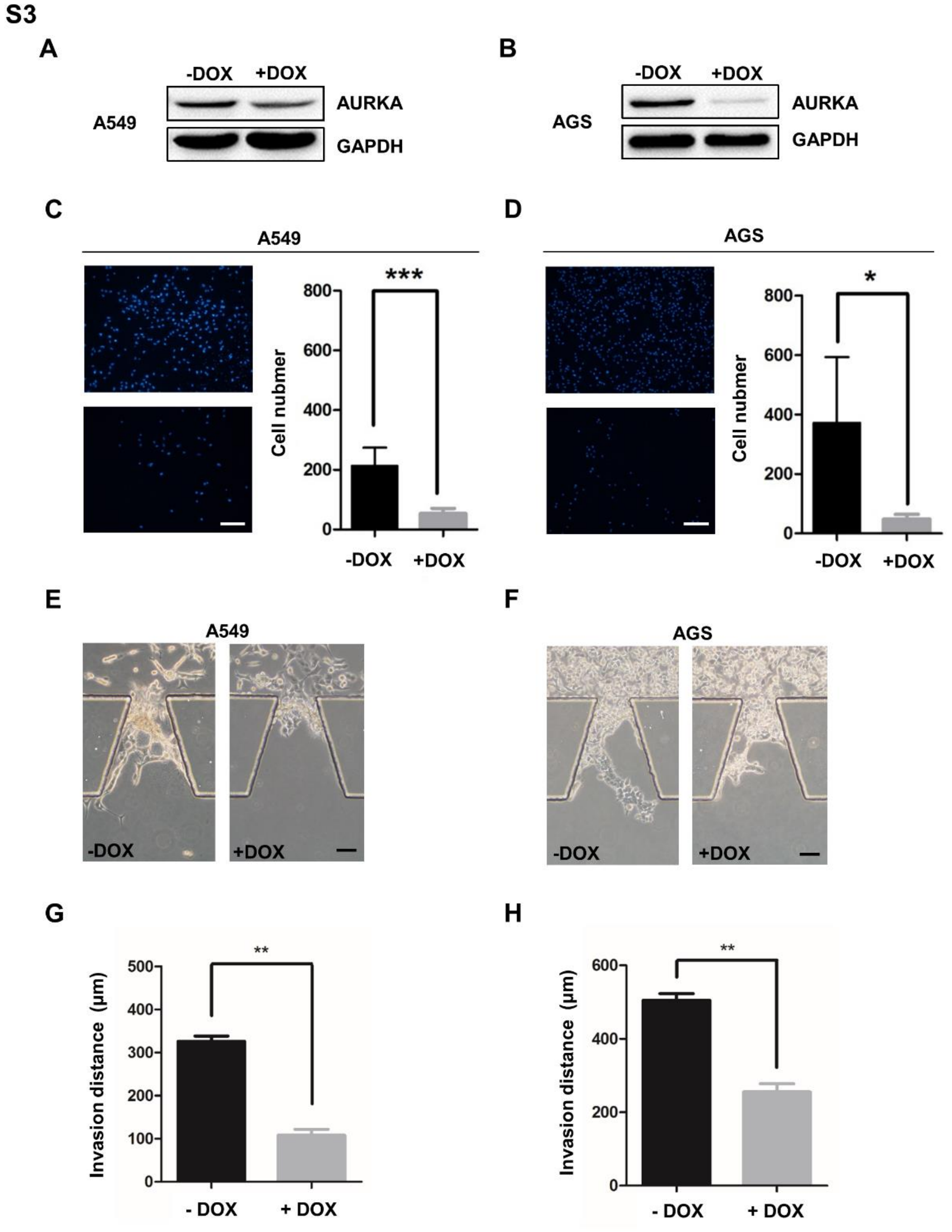

S3: Cell invasion was suppressed in A549 cells and AGS cells via knocking down AURKA. (A, B) Endogenous AURKA was conditional knocked down via treating with 0.2 μM doxycycline in A549$^{\text{tet-on shAURKA}}$ cells and AGS$^{\text{tet-on shAURKA}}$ cells. (C, D) Invasion of A549 cells and AGS cells was reduced via knocking down AURKA. For invasion assay, A549 cells AGS cells were seeded into the chamber of transwell

coated with 16% Matrigel. One representative of three independent experiments was shown. The nuclei were visualized by DAPI staining. Invasion rates were quantified by counting the invaded cells in five random fields. Data summarized three independent experiments (*, P < 0.05; ***, P < 0.001 Student t test). Scale bar: 100 μm. (E, F) A549 cells and AGS cells with or without doxycycline treatment were cultured in microfluidic chips for two days. Scale bar: 100 μm. (G, H) Invasion distance at day 2 were measured. Data summarized 10 duplicated galleries and were presented as the means ± SD (**, P < 0.01, Student t test).

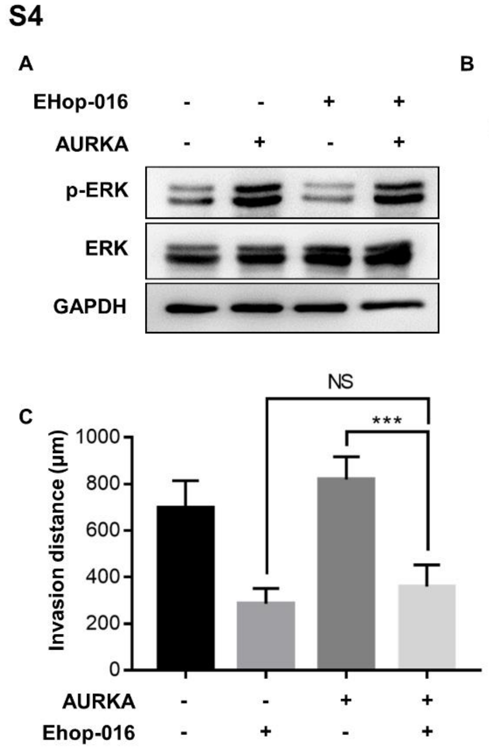

S4: AURKA promotes collective invasion via a Rac1-dependent manner. (A) MDA-MB-231 cells were transfected with control or AURKA vectors for 48hr and treated with or without Rac1 inhibitor Ehop-016 for a further 8hr. P-ERK was detected by western blot. (B) MDA-MB-231 cells were transfected with control or AURKA vector in combination with indicated inhibitor then seeded into microfluidic

device. Images were taken at day 0, day 2 respectively. (C) Invasion distance at day 2 were measured. (***, $P < 0.001$, two-way ANOVA). Scale bar: 100 μm.

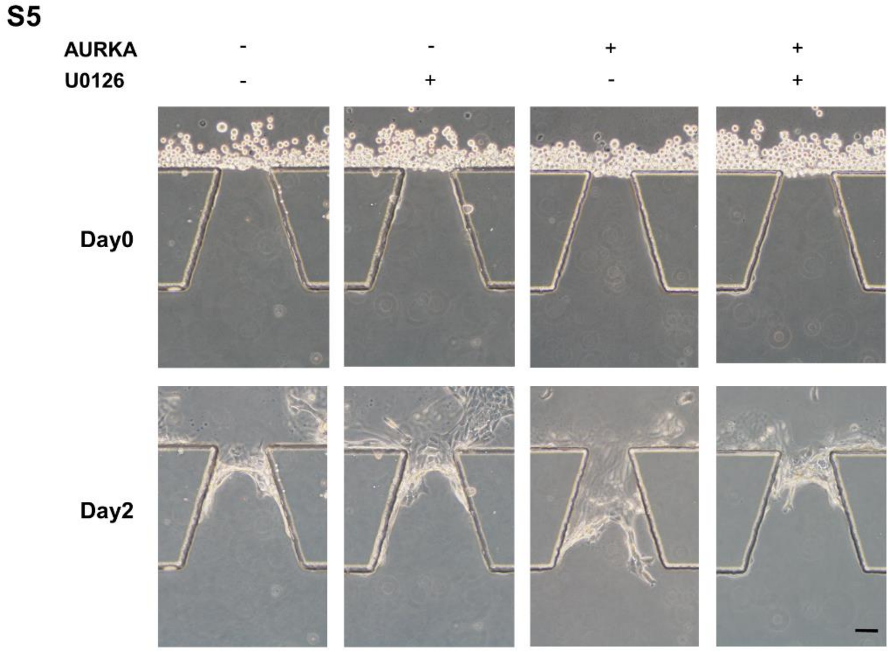

S5: Images of MCF-10A^{P} cells and MCF-10A$^{wt\ AURKA}$ cells cultured in microfluidic chip with or without U0126 treatment. MCF-10A^{P}, MCF-10A^{wtAURKA} cells and U0126 treated MCF-10AP and MCF-10Awt ARUKA cells were seeded in microfluidic chips for 2 days. Scale bar: 100 μm.

S6

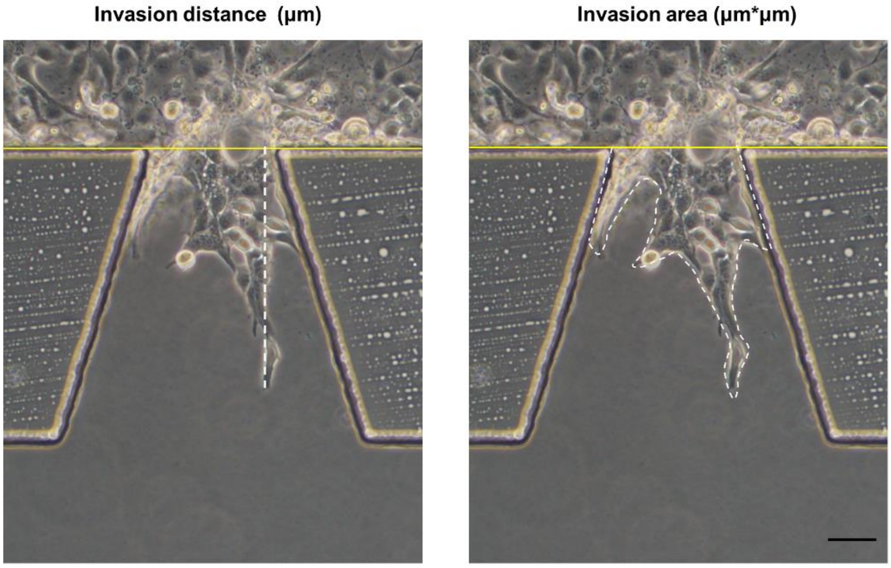

S6: Quantitative analysis of invasion distance and invasion areas. The invasion distance is defined as the distance between the starting line and the invasive edge of tumor cells. The invasion area is defined as the total space invading into the hydrogel. Scale bar: 100 μm.
